# Effect of Le Fort I osteotomy on the sinonasal region: three-dimensional study

**DOI:** 10.1016/j.bjorl.2026.101855

**Published:** 2026-06-18

**Authors:** Caio César Gonçalves Silva, Tatiane Fonseca Faro, Marcelo Soares dos Santos, Emanuel Dias de Oliveira e Silva, Jose Rodrigues Laureano Filho

**Affiliations:** a Universidade de Pernambuco, Faculdade de Odontologia, Campus Arcoverde, Arcoverde, PE, Brazil; b Universidade de Pernambuco, Faculdade de Odontologia de Pernambuco, Departamento de Cirurgia e Traumatologia Bucomaxilofacial, Recife, PE, Brazil

**Keywords:** Orthognathic surgery, LeFort osteotomy, Maxillary sinus, Nasal cavity, Cone-beam computed tomography

## Abstract

**Objective:**

The aim of the present study was to investigate changes in the maxillary sinus and nasal cavity in patients submitted to Le Fort I osteotomy using Cone Beam Computed Tomography (CBCT).

**Methods:**

A retrospective study was conducted involving the analysis of preoperative (T1) and postoperative (T2) CBCT scans of 34 patients undergoing orthognathic surgery. The outcome variables were sinus and nasal cavity volumes, presence and patency of the maxillary ostium, sinus mucosal thickening, nasal septal deviation, and changes in sinonasal anatomy. The paired-sample t-test and Wilcoxon test were used to compare differences between T1 and T2, with the significance level set at 5% (p < 0.05).

**Results:**

Age ranged from 18 to 55 years (mean: 29.97 ± 9.85). Most patients were women (64.7%) and had a type III facial profile (52.9%). Statistically significant differences were found between T1 and T2 in the mean volume of the maxillary sinus (p = 0.001) and nasal cavity (p = 0.042) and the degree of mucosal thickening of the maxillary sinuses (p = 0.013; p = 0.046).

**Conclusion:**

Le Fort I osteotomy is associated with significant changes in the volume of the maxillary sinus and nasal cavity as well as thickening of the sinus mucosa.

**Level of evidence:**

4.

## Highlights

• Discontinuity of the sinus walls, nasal cavity and nasal septum are common.
• Increased mucosal thickening of the maxillary sinuses in the postoperative period.
• Volumetric reduction of the maxillary sinus and nasal cavity in the postoperative.
• Monitoring of factors that may interfere with sinus homeostasis is suggested.

## Introduction

Le Fort I osteotomy is one of the most common techniques for the correction of midface deformities. In addition to orthognathic corrections, this method is also indicated for surgical access to structures at the base of the skull, in the treatment of cleft lip and palate, and in the correction of defects resulting from facial trauma. During the surgical procedure, osteotomy is performed from the pyriform aperture to the pterygomaxillary junction on both sides, making it possible to move the maxilla in three dimensions.^1^

The technique is so named due to its similarity to fracture pattern I described by Rene Le Fort in 1901. Le Fort I osteotomy has the advantages of technical ease and wide application, as it can solve a variety of functional and aesthetic problems.^2^ However, the surgical movements of this procedure can cause changes in the airway space and maxillary sinus.^3^

Recent advances in surgical techniques have reduced the number of complications associated with osteotomy and it is considered a safe, reliable, predictable procedure.^2^ In Le Fort I osteotomy, however, the bone surrounding the maxillary sinus is separated, leading to blood pooling in the sinus and an inflammatory process in the sinus mucosa. The risk of maxillary sinusitis is high if the blood pool or mucosal thickening persists for an extended period of time.^4^

The effects of orthognathic surgery on the anatomy and physiology of the upper airway have been extensively studied. Maxillomandibular advancement is associated with increases in the volume of the upper airway and upper pharynx, with a notable improvement in airflow. In contrast, studies investigating the effects of orthognathic surgery on the sinonasal region are less common and offer conflicting results.^4^, ^5^, ^6^, ^7^, ^8^, ^9^

Therefore, the aim of the present study was to investigate changes in the sinonasal region in patients submitted to Le Fort I osteotomy using Cone Beam Computed Tomography (CBCT), with an analysis of the occurrence of anatomical defects in the nasal cavity and maxillary sinus in the preoperative period and surgically induced changes in the postoperative period.

## Methods

### Study design

A retrospective study was conducted involving the tomographic analysis of the sinonasal region in patients submitted to Le Fort I osteotomy. This study was reported in accordance with the Strengthening the Reporting of Observational Studies in Epidemiology (STROBE statement)^10^ and received approval from the institutional review board of the Research Ethics Committee of the University of Pernambuco under opinion number 4.322.222.

### Patients

The universe of the present study is comprised of patients who underwent orthognathic surgery at the Oswaldo Cruz University Hospital (HUOC), Recife-Pernambuco, Brazil, from January 2015 to June 2022. Patients over 18-years of age who underwent Le Fort I osteotomy alone or in combination with jaw osteotomy and had clinical and tomographic information recorded in the pre- and postoperative periods were included in the study. Patients with craniofacial anomalies, previous paranasal surgeries, a history of associated disease or treatment with medication that affected bone quality, and a history of multiple orthognathic surgical procedures were excluded.

### Image acquisition

CBCT images were acquired at two timepoints: before surgery (T1) and between three and twelve months after surgery (T2), in accordance with the protocol of the Oral and Maxillofacial Surgery team at HUOC. The images were converted into Digital Imaging and Communications in Medicine (DICOM) files and exported to the Dolphin Imaging 12.0 software (Dolphin Imaging and Management Solutions, Chatsworth, CA).

### Surgical procedure

A single oral-maxillofacial surgeon (JRLF) and his team performed the operation on all patients under general anesthesia with nasotracheal intubation. Le Fort I osteotomy was performed with a reciprocating saw. To avoid deviation of the nasal septum after surgery, the nasal septal cartilage, vomer, and lateral wall of the nasal cavity were carefully separated from the maxilla with chisels. When fracturing the maxilla, the septum and floor of the nose were exposed, and the septal cartilage was resected with blunt scissors. Osteoplasty was performed on the superior crest and medial wall of the maxillary sinuses to avoid interference during maxillary repositioning. In cases of damage, the nasal mucosa was repaired with resorbable suture. Functionally stable internal fixation with titanium plates and screws was performed in all cases.

### Outcome measures

Data were collected on demographic (age and sex), clinical (facial profiles I, II, and III),^11^^,^^12^ and surgical (extent and direction of the planned movement of the maxilla after Le Fort I osteotomy, taking into account the position of the upper central incisor) characteristics.

**Fig. 1:**
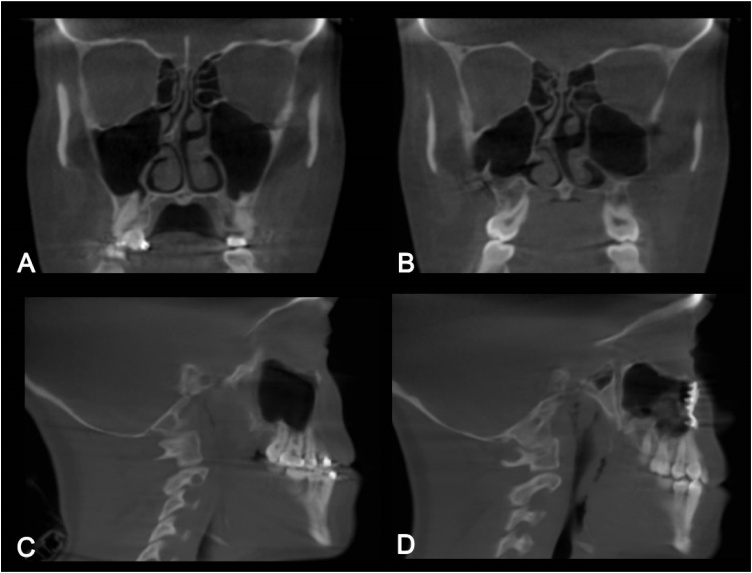
Anatomy of sinonasal region. (A) CT image (coronal section) of sinonasal region in preoperative period; (B) CT image (coronal section) of sinonasal region in postoperative period. (C) CT image (sagittal section) of sinus region in preoperative period; (D) CT image (sagittal section) of sinus region in postoperative period.

The tomographic analysis was performed by a previously calibrated examiner (weighted Kappa Test = 0.948; intraclass correlation coefficient = 0.998), using the Dolphin Imaging 12.0 software to perform three-dimensional analysis as well as linear and angular measurements. The images were analyzed in coronal, axial, and sagittal sections, with the investigation of anatomical aspects of the sinonasal region and changes induced by orthognathic surgery (Fig. 1). The primary outcome variables were the volumes of the sinus and nasal cavities. The secondary outcome variables were the presence and patency of the maxillary ostium, thickening of the sinus mucosa, deviation of the nasal septum, and iatrogenic changes to sinonasal anatomy.

**Fig. 2:**
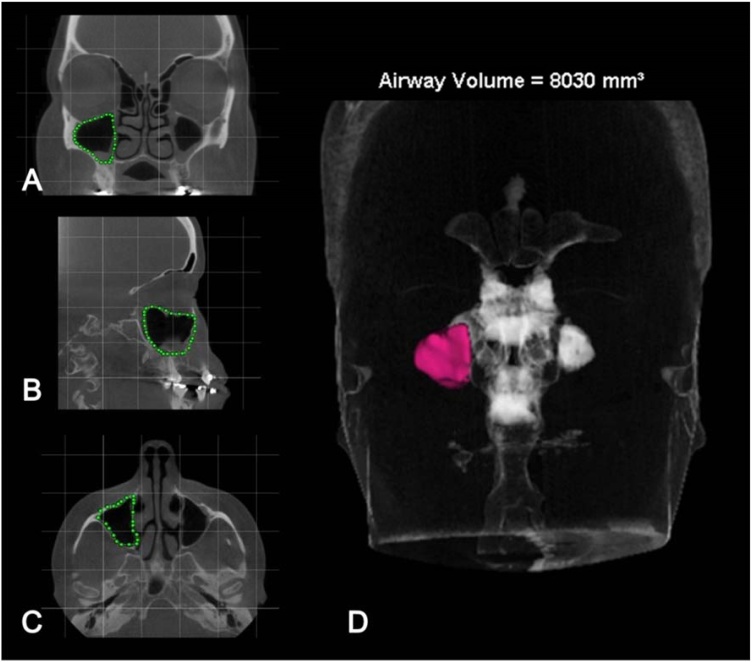
Volumetric analysis of right maxillary sinus. (A, B, and C) Manual delimitation in coronal, sagittal, and axial sections, respectively. (D) Determination of volume of right maxillary sinus using airway analysis tool of Dolphin Imaging software.

The airway analysis tool of the Dolphin Imaging software was used for the volumetric analysis of the maxillary sinus. The examiner manually delimited the area corresponding to the maxillary sinus. The tool for selecting only pixels within a predefined range of Hounsfield Units (HU) (minimum: -1024; maximum: 600) was then used to select pixels considered to be the maxillary sinus in the tomographic images. The selected pixels were remodeled into three-dimensional images and the volumes of the right and left sinuses were calculated separately, as shown in Fig. 2.^7^^,^^13^^,^^14^

**Fig. 3:**
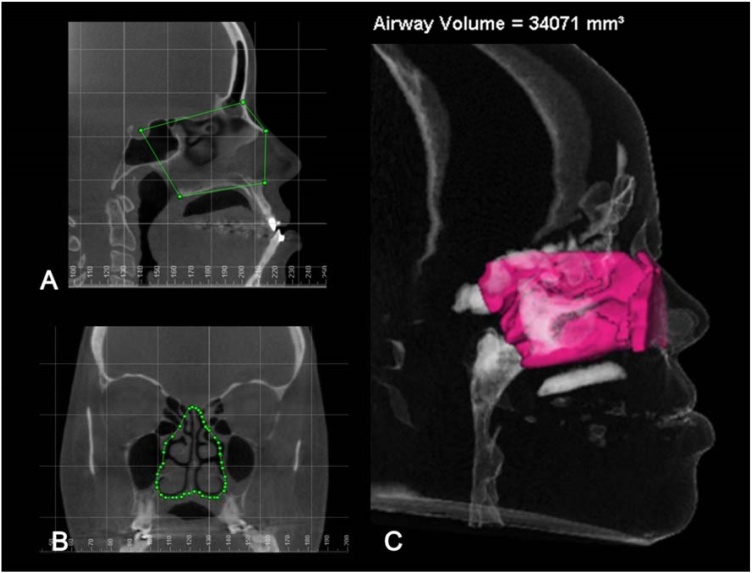
Volumetric analysis of nasal cavity. (A and B) Manual delimitation in sagittal and coronal sections, respectively. (C) Determination of volume of nasal cavity using airway analysis tool of Dolphin Imaging software.

A similar procedure was used for the volumetric analysis of the nasal cavity. Manual delimitation of the area in the sagittal section took into account the following anatomical points based on the study by Smith et al.^15^: the anterior limit was a line connecting the Anterior Nasal Spine (ANS) to the tip of the nasal bone and the Nasion (N); the posterior limit was a line extending from the Sella point (S) to the Posterior Nasal Spine (PNS); the upper limit was a line joining the N point to S; the lower limit was a line extending from the ANS to the PNS. In the coronal section, manual delimitation was performed around the bony structure that borders the nasal cavity (Fig. 3). The airway analysis tool of the Dolphin Imaging software was then used to calculate the volume of the nasal cavity.^14^

**Fig. 4:**
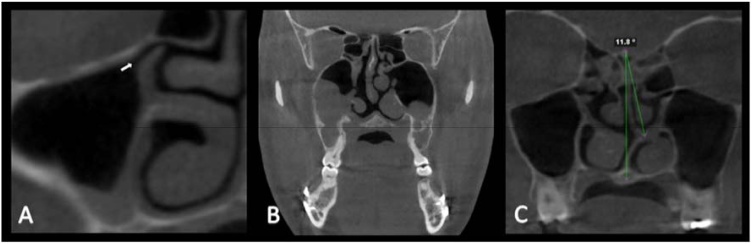
(A) CT image (coronal section) showing position of right maxillary ostium. (arrow). (B) CT image (coronal section) showing thickening of maxillary sinus mucosa bilaterally. (C) CT image (coronal section) showing nasal septum deviation using angular measurement tool of Dolphin Imaging software.

The presence or absence of the maxillary ostium was determined (Fig. 4A). If present, the patency of the maxillary ostium was assessed (obstructed or patent). The presence of accessory maxillary ostia was also investigated.^16^

The measurement of the mucosal thickness was performed in both maxillary sinuses and took into account the point of greatest mucosal thickening on each side, as shown in Fig. 4B. Thickening was classified as Grade I (thickening occupies up to 1/3 of the maxillary sinus), Grade II (thickening occupies 1/3 to 2/3 of the maxillary sinus), and Grade III (thickening occupies more than 2/3 of the maxillary sinus).^4^

Fig. 4C shows the measurement of the angle of deviation of the nasal septum, which was performed through the coronal cut using a line connecting the crista Galli to the anterior nasal spine and a second line joining the crista Galli to the most deviated point of the nasal septum. After marking the reference points, the angle measurement tool of the Dolphin Imaging software was used. For “S”-shaped septa, the largest angle was considered. The angle of nasal septal deviation was classified as mild (<9°), moderate (9°–15 °), or severe (>15 °).^17^, ^18^, ^19^

### Statistical analysis

The database of the study was built with the aid of the Statistical Package for Social Sciences (SPSS® software, version 20.0.0). The variables were treated using descriptive statistics, with the calculation of absolute and relative frequencies and measures of central tendency and dispersion. The Shapiro-Wilk test was used to determine the distribution of the data, which demonstrated normal distribution. The mean volumes of the maxillary sinuses and nasal cavity in periods T1 and T2 were compared using the paired-sample t-test, whereas ordinal variables were compared using the Wilcoxon test. Volumetric outcome variables were categorized dichotomously as “increase” and “decrease” and associations with the predictor variables were investigated using the Chi-Square test. The significance level was set at 5% (p < 0.05) for all statistical tests.

## Results

The sample consisted of 34 individuals, totaling 68 tomographic exams. Age ranged from 18 to 55-years (mean: 29.97 ± 9.85 years) and most patients were women (64.7%). The frequency of the facial profile classifications according to Arnett and Bergman^10^^,^^11^ was 2.9% for type I, 44.1% for type II, and 52.9% for type III.

In the preoperative tomographic analysis of the anatomy of the maxillary sinus, a mucous retention cyst was found in two patients (5.9%), whereas the others did not exhibit any anatomical abnormalities. In the postoperative period, bone fixation screws were observed in the maxillary sinus in all patients and discontinuity of the maxillary sinus wall was found in two patients (5.9%). In the preoperative tomographic analysis of the anatomy of the nasal cavity, turbinate hypertrophy was found in two patients (5.9%) and marked deviation of the nasal septum was found in 7 (20.5%). In the postoperative assessment, discontinuity of one or more walls of the nasal cavity was found in 27 cases (79.4%), discontinuity of the nasal septum was found in two patients (5.9%), and marked deviation of the nasal septum was found in eight (23.5%).

Table 1 shows the variations in sinonasal characteristics between the T1 and T2 tomographic exams. Statistically significant reductions were found in the mean volumes of the maxillary sinus (p = 0.001) and nasal cavity (p = 0.042) between T1 and T2. Moreover, a significant increase occurred in the degree of mucosal thickening of the right (p = 0.013) and left (p = 0.046) maxillary sinuses between T1 and T2. No significant differences were found with regards to the presence and patency of the maxillary ostium (right: p = 0.317; left: p = 0.527) or degree of nasal septal deviation (p = 0.206) between T1 and T2. Moreover, no accessory maxillary ostia were identified in the tomographic exams.

**Table 1:** Sinonasal characteristics in pre- and postoperative tomographic exams.

	T1		T2		
Volume of maxillary sinuses^a^	mm^3^		mm^3^		p-value
Interval	6095.00 – 32382.50		6073.00 – 34032.00		0.001
Mean ± SD	20252.22 ± 6041.06		17162.48 ± 7453.16		
**Volume of nasal cavity**	**mm^3^**		**mm^3^**		**p-value**
Interval	18266.00 – 54064.00		19224.00 – 44257.00		0.042
Mean ± SD	34532.85 ± 7906.00		31311.06 ± 7230.72		
**Right maxillary ostium**	**n**	**%**	**n**	**%**	**p-value**^b^
Present – patent	32	94.1	30	88.2	0.317
Present – obstructed	1	2.9	3	8.8	
Absent	1	2.9	1	2.9	
**Left maxillary ostium**	**n**	**%**	**n**	**%**	**p-value**^b^
Present – patent	32	94.1	29	85.3	0.527
Present – obstructed	1	2.9	5	14.7	
Absent	1	2.9	0	0	
**Mucosal thickening of right maxillary sinus**	**n**	**%**	**n**	**%**	**p-value**^b^
Grade I	31	91.2	23	67.6	0.013
Grade II	2	5.9	7	20.6	
Grade III	1	2.9	4	11.8	
**Mucosal thickening of left maxillary sinus**	**n**	**%**	**n**	**%**	**p-value**^b^
Grade I	33	97.1	27	79.4	0.046
Grade II	0	0	4	11.8	
Grade III	1	2.9	3	8.8	
**Deviation of nasal septum**	**n**	**%**	**n**	**%**	**p-value**^b^
Mild	15	44.1	12	35.3	0.206
Moderate	12	35.3	14	41.2	
Severe	7	20.6	8	23.5	
**Total**	**34**	**100.0**	**34**	**100.0**	

a *t*-test for paired samples.

b Wilcoxon signed rank test.

No statistically significant associations were found between the volumetric change in the maxillary sinuses/nasal cavity and sex, facial profile, and planned surgical movement of the maxilla (Table 2).

**Table 2:** Volumetric variation in maxillary sinuses and nasal cavity according to sex, facial profile, and planned surgical movement of maxilla.

	Maxillary sinus volume				Nasal cavity volume			
	Increase, n (%)	Reduction, n (%)	χ^2^	p-value	Increase, n (%)	Reduction, n (%)	χ^2^	p-value
**Sex**								
Female	16 (72.7%)	6 (27.3%)	0.735	0.315	16 (72.7%)	6 (27.3%)	0.137	0.502
Male	7 (58.3%)	5 (41.7%)			8 (66.7%)	4 (33.3%)		
**Facial profile**								
Type I	1 (100%)	0 (0%)	2,727	0.256	1 (100%)	0 (0%)	4.244	0.120
Type II	12 (80%)	3 (20%)			13 (86.7%)	2 (13.3%)		
Type III	10 (55.6%)	8 (44.4%)			10 (55.6%)	8 (44.4%)		
**Vertical movement**								
Impaction	11 (78.6%)	3 (21.4%)	2,516	0.284	11 (78.6%)	3 (21.4%)	1,453	0.484
Extrusion	8 (53.3%)	7 (46.7%)			9 (60%)	6 (40%)		
No movement	4 (80%)	1 (20%)			4 (80%)	1 (20%)		
**Anteroposterior movement**								
Advance	21 (67.7%)	10 (32.3%)	0.763	0.683	22 (71%)	9 (29%)	0.827	0.661
Retreat	1 (50%)	1 (50%)			1 (50%)	1 (50%)		
No movement	1 (100%)	0 (0%)			1 (100%)	0 (0%)		
**Lateral-lateral movement**								
Shift to right	7 (70%)	3 (30%)	1,399	0.497	5 (50%)	5 (50%)	5.252	0.072
Shift to left	10 (76.9%)	3 (23.1%)			12 (92.3%)	1 (7.7%)		
No movement	6 (54.5%)	5 (45.5%)			7 (63.6%)	4 (36.4%)		
**Total**	**23 (**67.6%**)**	**11 (**32.3%**)**	**‒**	**‒**	**24 (**70.5%**)**	**10 (**29.4%**)**	**‒**	**‒**

## Discussion

Although several studies^2^^,^^20^, ^21^, ^22^, ^23^, ^24^ have assessed complications associated with Le Fort I osteotomy, reports on sinonasal changes in patients submitted to this procedure are lacking.

The analysis of the preoperative anatomy revealed sinonasal abnormalities, such as mucous retention cysts, marked deviation of the nasal septum, and turbinate hypertrophy in a small percentage of patients. Although not common, such conditions may play a role in the onset of rhinosinusitis. This possibility should be considered during surgical planning and auxiliary procedures may be adopted, such as septoplasty in cases of marked deviation of the nasal septum and turbinectomy in situations of turbinate hypertrophy.^7^^,^^25^

Surgically induced alterations were found in a substantial percentage of patients in this study. The most frequent were discontinuities of the nasal septum, sinus wall, and nasal cavity. Moreover, bone fixation screws were observed in the maxillary sinus of all patients, as also reported in the study conducted by Nocini et al.^7^ Although this situation is unavoidable in most cases, the association between screw infection and alterations in sinonasal anatomy may compromise the homeostasis of the sinus and contribute to the pathogenesis of severe sinusitis.^8^ Short screws can be used to avoid a potential contributing factor to the disruption of homeostasis.

The nasal septum is an important structure in the anatomy of the nose. Deviation from the midline can affect both aesthetics and function.^26^^,^^27^ Although maxillary movement during orthognathic surgery has the potential to alter the nasal septum,^27^ changes in the degree of septal deviation after surgery were not statistically significant, which is in agreement data described in previous studies.^9^^,^^14^^,^^26^^,^^28^^,^^29^ Most patients with marked deviation of the nasal septum had this condition both pre- and postoperatively. A possible reason for deviation of the cartilaginous septum after maxillary osteotomy is given in the study conducted by Acebal-Bianco et al.,^30^ who describe displacement of the nasal septum by a partially deflated cuff during extubation. This underscores the importance of the manual inspection of the nostrils after extubation via the nasotracheal route.

The maxillary ostium is the communication between the sinus and nasal cavities, and its presence and patency are indicative of good maxillary sinus function.^16^ Injury to the antral alveolar artery may occur in Le Fort I osteotomy, with the possibility of rupture of the sinus membrane and a reduction in the permeability of ostium, thus increasing the likelihood of postoperative sinusitis.^31^, ^32^, ^33^, ^34^ In the present study, no statistically significant differences were found with regards to the presence and patency of the maxillary ostia between the pre- and postoperative assessments.

Another variable of interest was the thickening of the maxillary sinus mucosa after Le Fort I osteotomy. A significant increase was found in the degree of mucosal thickening of the maxillary sinuses at T2. Studies conducted by Baeg et al.,^14^ Iwamoto et al.,^4^ and Akbulut et al.^35^ report similar results. The bone surrounding the maxillary sinus is separated during osteotomy, causing the accumulation of blood in the sinus cavity, with thickening of the mucosa. This condition may be a precursor to an inflammatory process, with negative impacts on the ciliary activity of the mucosa and drainage of the sinus.^4^

Toskala and Rautiainen^36^ assessed the maxillary sinus mucosa after surgery and reported that pathological conditions persisted up to six months postoperatively. Therefore, frequent follow-up is required for a period of at least one year after surgery. The increased degree of mucosal thickening in the present study may be explained by the time of postoperative tomographic image acquisition (T2), which occurred prior to six months in most patients.

The volume of the maxillary sinus after Le Fort I osteotomy has been measured by some authors,^7^^,^^14^^,^^35^^,^^37^^,^^38^ who are unanimous in stating that this surgical procedure causes a volumetric decrease in the maxillary sinuses. In the present study, although most patients exhibited an increase in the volume of the maxillary sinus after Le Fort I osteotomy, the mean total volume of the maxillary sinus was significantly reduced after surgery (p = 0.001). This finding is in agreement with results described in studies conducted by Nocini et al.,^7^ Baeg et al.,^14^ and Akbulut et al.^35^ The volumetric change is related to the thickening of the sinus membrane. Therefore, the time of postoperative image acquisition may also have influenced this result. A follow-up period longer than six months is necessary for more accurate volumetric measurements of the maxillary sinus.

Studies have used different methods to assess volumetric changes in the nasal cavity of patients submitted to Le Fort I osteotomy.^26^^,^^39^, ^40^, ^41^ In most of these studies, a volumetric reduction in the nasal cavity occurred in the postoperative period. This result was also found in the present study, in which the mean reduction in volume was 3,221.79 mm^3^, although a greater number of patients exhibited an increase in the volume of the nasal cavity after Le Fort I osteotomy. As described in the study conducted by Erbe et al.,^39^ a volumetric reduction in the nasal cavity does not always have a negative impact on respiratory function.

Despite the statistically significant volumetric reductions in the maxillary sinus and nasal cavity, no associations were found between these changes and sex, facial profile, or planned surgical movement of the maxilla. Future studies with larger samples may find associations between volumetric changes and some variables of interest.

The findings of the present study suggest that clinical and imaging monitoring of patients undergoing Le Fort I osteotomy should focus not only on the occlusal plane and postsurgical stability of the maxilla, but also on factors that may interfere with the homeostasis of the sinus.

Certain limitations of this study should be taken into account when interpreting the findings. There was no standardization of T2, which ranged from three to twelve months. The wide variation in the timing of postoperative CT scans should be considered a significant methodological limitation, as it can directly influence the interpretation of the results. Residual inflammation, edema, and thickening of the sinus mucosa are more frequent in earlier periods, which can overestimate volumetric changes or mask anatomical stabilization. These changes tend to regress in later CT scans, enabling a more reliable analysis of the morphofunctional adaptation of the maxillary sinus. Therefore, temporal heterogeneity between exams can introduce bias in the comparison of the findings, interfering with the consistency and reproducibility of the data obtained.

The lack of correlation between clinical sinonasal data and CT scans due to the retrospective nature of this study is another limitation and makes it difficult to extrapolate the results to clinical practice. Although CT scans enable the identification of mucosal thickening, volumetric changes in the maxillary sinus and nasal cavity, and nasal septum deviation, the images do not necessarily translate into clinical symptoms, such as sinusitis, nasal obstruction, or respiratory distress. Therefore, the lack of clinical information impedes the determination of whether the CT scans have functional relevance or a direct impact on quality of life.

Well-conducted prospective clinical trials with pre- and postoperative clinical and imaging analyses, a larger sample, and standardized data collection are needed to assess the clinical impact of Le Fort I osteotomy in the sinonasal region and, consequently, on the functionality of the maxillary sinus and nasal cavity.

## ORCID ID

Caio César Gonçalves Silva: 0000-0002-7519-7894

Tatiane Fonseca Faro: 0000-0002-9389-2567

Marcelo Soares dos Santos: 0000-0001-7262-6035

Emanuel Dias de Oliveira e Silva: 0000-0003-1010-704X

Jose Rodrigues Laureano Filho: 0000-0002-9645-2057

## Conclusion

The tomographic analysis of the sinonasal region of patients submitted to Le Fort I osteotomy suggests a reduction in the mean volume of the maxillary sinus and nasal cavity as well as an increase in the degree of mucosal thickening of the maxillary sinuses in the postoperative period.

## Funding

This study was funded by the Coordenação de Aperfeiçoamento de Pessoal de Nível Superior – Brasil (CAPES) – Finance code 001.

## Declaration of competing interest

The authors declare no conflicts of interest.
